# Habenula volume alterations in adults born very preterm

**DOI:** 10.1038/s41598-026-61917-5

**Published:** 2026-07-14

**Authors:** Theresa Stüwe, Angelika Maurer, Marcel Daamen, Barbara D. Wichtmann, Hyun Soo Ko, Neeraj Upadhyay, Benita Schmitz-Koep, Markus Essler, Julian Luetkens, Claus Zimmer, Peter Bartmann, Dieter Wolke, Dennis Hedderich, Christian Sorg, Henning Boecker

**Affiliations:** 1 University of Bonn, University Hospital Bonn, Department of Nuclear Medicine, Clinical Functional Imaging Group, Bonn, Germany; 2 University of Bonn, University Hospital Bonn, Department of Diagnostic and Interventional Radiology, Bonn, Germany; 3 University of Bonn, University Hospital Bonn, Department of Neonatology and Pediatric Intensive Care, Bonn, Germany; 4 University of Bonn, University Hospital Bonn, Clinic of Neuroradiology, Bonn, Germany; 5 University of Bonn, University Hospital Bonn, German Center for Neurodegenerative Diseases (DZNE), Bonn, Germany; 6 Department of Cancer Imaging, Peter MacCallum Cancer Centre, Melbourne, Australia; 7 The Sir Peter MacCallum Department of Oncology, University of Melbourne, Melbourne, Australia; 8 Department of Neuroradiology, School of Medicine and Health, TUM Klinikum rechts der Isar, Technical University of Munich, Munich, Germany; 9 TUM-NIC Neuroimaging Center, School of Medicine and Health, TUM Klinikum rechts der Isar, Technical University of Munich, Munich, Germany; 10 Department of Psychology, University of Warwick, Coventry, UK; 11 Warwick Medical School, University of Warwick, Coventry, UK; 12 Department of Psychiatry, School of Medicine and Health, TUM Klinikum rechts der Isar, Technical University of Munich, Munich, Germany; 13 University of Bonn, University Hospital Bonn, Department of Nuclear Medicine, Bonn, Germany

**Keywords:** Structural MRI, Neuroradiology, Anxiety, Depression, Avoidant personality, Bavarian, Longitudinal study, Diseases, Health care, Medical research, Neuroscience, Psychology, Psychology

## Abstract

Preterm birth is a major risk factor for childhood psychopathology, including higher rates of anxiety and avoidant personality traits persisting into adulthood. While extensive research has documented volumetric reductions in various brain structures associated with emotional processing in preterm individuals, the habenula, a key component in affective and reward processing, has not yet been studied in this context. This study examines whether adults born very preterm show alterations in the habenula volume and whether these alterations are linked to higher anxiety, depression and avoidant personality traits. This study utilized data from the Bavarian Longitudinal Study, comprising 103 preterm-born individuals (defined as very preterm (VP) < 32 weeks of gestational age and/or very low birth weight (VLBW) <1500 g) and 110 term-born (TB) controls. Habenula volumes were assessed using manual segmentation on T1-weighted MRI scans. Additionally, participants completed standardized psychological assessments measuring anxiety, depression, and avoidant personality traits (Achenbach Young Adult Self-Report (YASR) and Beck Depression Inventory ). A higher relative right habenula volume was observed at trend-level in the VP sub-sample when compared to TB controls (Bonferroni-corrected p = 0.080). VP/VLBW showed a significant positive correlation between the relative right habenula volume with intensity of neonatal treatment. Additionally, VP and VP/VLBW exhibited higher scores on YASR anxiety and avoidant personality scales when compared to TB controls, yet were not correlated with habenula volumes. Our findings indicate that very preterm birth is associated with a trend towards relatively increased right habenula volume and higher levels of anxiety and avoidant personality traits in adulthood. While habenula volume was not directly linked to these traits, its association with intensity of neonatal treatment suggests a genuine role of perinatal stress on adult habenular morphology.

**Supplementary Information:**

The online version contains supplementary material available at 10.1038/s41598-026-61917-5.

## Introduction

Preterm birth is a significant global health concern, affecting approximately 10% of all newborns^1^. Advances in neonatal care have significantly improved survival rates in the last decades, particularly for neonates classified as very preterm (VP,<32 weeks of gestational age) and very low birth weight (VLBW,<1500 g)^2^. This has led to increased research on the long-term consequences of preterm birth to ensure appropriate medical and psychological care for former preterm individuals.

Preterm birth is increasingly acknowledged as one of the most prevalent risk factors for childhood psychopathology as VP/VLBW frequently exhibit higher levels of anxiety disorders and avoidant personality traits in adolescence and adulthood^3,4^. These psychiatric vulnerabilities co-occur with well-documented alterations in brain structure. Volumetric reductions have been observed in several brain regions, including the thalamus, hippocampus, amygdala, corpus callosum, and basal ganglia^5^. These regions are implicated in a wide range of neural functions, some of which relate to affective processing. Notably, abnormalities in these regions have been associated with psychiatric conditions involving affective dysregulation^6,7^, e.g. amygdala^8^.

The habenula is an important hub region known for its role in modulating affective and motivational processes and functionally linked to several psychiatric disorders. The habenula is a bilateral epithalamic structure located between the dorsomedial thalamus and the third ventricle, rostral to the posterior commissure. It consists of the medial and lateral habenula, receiving afferent input via the stria medullaris thalami and projecting efferent signals through the retroflex fascicle, also known as the habenulointerpeduncular tract. The habenula is involved in various functions, including reward behavior^9,10^, pain processing^11^, reproductive behavior^12^, sleep-wake cycles^13,14^, stress responses, learning^15^ and processing aversive stimuli^16,17^. Emerging research underscores the habenula’s involvement in several psychiatric disorders, especially depression and anxiety^18–20^. As a central hub within the dorsal diencephalic conduction system, the habenula integrates basal ganglia and limbic system inputs, modulating midbrain structures. This modulation involves a variety of neurotransmitters, including serotonin, dopamine, GABA, glutamate, and acetylcholine. This highlights the habenula’s crucial role in regulating the monoaminergic system, positioning it as a unique key structure in neurophysiological processing^21^.

In humans, lateralised habenular function has been demonstrated in both resting-state and task-based fMRI. High-resolution resting-state data show that left and right habenula are only weakly correlated (*r* < 0.10) and exhibit distinct connectivity profiles, with stronger coupling of the right habenula to the substantia nigra and ventral tegmental area and stronger coupling of the left habenula to limbic and cortical regions, suggesting that the two nuclei may serve partially distinct functional roles^22^.

Task-based studies of aversive learning further indicate functional asymmetry between the two habenular nuclei. In healthy adults, the left habenula responds more strongly to punishment outcomes than the right^23^, a pattern consistent with broader findings of asymmetric habenular activation during aversive processing^16,24^.

At the structural level, the nature of left-right differences in habenular volume remains debated. A large-scale meta-analysis of 52 datasets (*N* = 1,427) found significant heterogeneity across studies, with altered asymmetry patterns reported in psychiatric disorders: a trend towards leftward asymmetry (left > right) was observed in major depressive disorder, whereas schizophrenia showed a trend towards opposite, rightward asymmetry (left < right); however, neither difference reached statistical significance relative to healthy controls^25^.

Given the habenula’s critical role in neuropsychiatric diseases and the increased prevalence of mood disorders in preterm individuals, preterm birth might affect adult habenula volume, impacting the vulnerability for psychiatric dysfunctions. To date, no studies have addressed this question. Here we aim to fill this gap by comparing habenula volume between preterm and term-born (TB) individuals using voxel-based morphometry of T1w MRI scans, also considering potential hemispheric differences. Following recommendations from previous studies^26–28^, we focused on gestational age (GA) as primary indicator of prematurity and conducted separate analyses within the VP subgroup, excluding VLBW-only individuals due to their greater heterogeneity, including higher rates of small-for-gestational-age births and distinct perinatal risk profiles.

## Materials and methods

This MRI study is part of the prospective Bavarian Longitudinal Study, a geographically defined whole-population sample of VP/VLBW and TB individuals. Participants were followed from birth into early adulthood to examine their developmental status^29,30^. Therefore, neurological and psychological test batteries, as well as parental interviews during childhood, adolescence, and at 26 years of age, were repeatedly assessed. Following the behavioral assessments in adulthood, eligible participants were invited for an additional MRI examination on a separate occasion.

### Participants

Initial recruitment took place in Phase 1, in which all infants admitted to one of 19 neonatal units (across 17 hospitals) in Southern Bavaria within the first 10 days of life were considered for participation. A total of 7505 newborns (10.6% of all live births) were enrolled, including 2759 preterm infants born before 37 weeks of gestation. Of these preterm infants, 682 were classified as VP and/or VLBW. The control group consisted of 916 TB infants recruited from the same hospitals. TB newborns were randomly selected to serve as the matched control group, based on the stratification variables gender and family socioeconomic status, ensuring comparability to the VP/VLBW group.

Participants have been followed prospectively from birth onward. For 26-year assessments, 411 VP/VLBW were eligible, with 260 (63.3%) participating in psychological assessments^31^. Among 308 eligible TB, 229 (74.4%) participated in psychological assessments. For the present analyses, only data from participants with additional MRI data from this 26-year follow-up were used. All were screened for MRI-related exclusion criteria, including claustrophobia, medical instability, epilepsy, tinnitus, pregnancy, MRI-incompatible implants, and severe CNS conditions. However, the most common reason for not undergoing MRI was that participants declined participation. In total, 103 VP/VLBW and 110 TB individuals were included in MR data processing. Of the 103 VP/VLBW, 82 were classified as VP.

### Background characteristics

GA was estimated based on maternal reports of the first day of the last menstrual period and serial ultrasounds during pregnancy. If these two measures differed by more than two weeks, GA was assessed clinically at birth using the Dubowitz method^32^.

Maternal age, birth weight (BW), and the duration (DNTI, Duration of Neonatal Treatment Index) and intensity (INTI, Intensity of Neonatal Treatment Index), which quantify the duration and extent of medical interventions after birth, were extracted from the neonatal records^29,33^.

As part of the MRI assessment, the Edinburgh Handedness Inventory (EHI) was administered. The EHI is a widely used self-report scale designed to assess an individual’s handedness, categorizing them as left-handed (EHI: −100 to − 61), right-handed (EHI: +61 to + 100), or ambidextrous (EHI: −60 to + 60) based on their preference for various hand-related tasks^34^. The inventory consists of 10 items, where participants rate their hand preference for activities such as writing, drawing, and using tools^35^.

### Assessment of affective symptoms in adulthood

Depressive and anxiety symptoms, along with psychiatric diagnoses, were comprehensively evaluated as part of the 26-year behavioral assessments. We focused on the assessments of psychological symptoms from the Achenbach Young Adult Self-Report (YASR)^36^, a standardized tool for measuring behavioral and emotional problems. The YASR consists of 116 items rated on a 3-point scale, generating subscale scores for internalizing problems (‘anxious/depressed’ and ‘withdrawn’ subscales) and externalizing problems (‘aggressive behavior,’ ‘rule-breaking behavior,’ and ‘intrusive behavior’). A detailed description can be found in Ni et al.^37^. Here, we employed the DSM-oriented scales for anxiety, depression and avoidant personality to improve concordance between Achenbach’s internalizing scales and DSM-IV classifications^8^. For all YASR subscales, T-standardized scores were used to facilitate comparison with normative data.

Further, the Beck Depression Inventory (BDI), a validated self-report questionnaire^38^ was applied on the MR examination day to assess depressive symptom severity across cognitive, emotional, and physical dimensions, with higher scores indicating greater symptom severity.

Given research linking habenula hyperactivity to depression^20^, this study focused on the BDI and the YASR DSM-oriented scales for anxiety, depression and avoidant personality.

### MRI data acquisition

MRI examinations were conducted at two sites: the Department of Neuroradiology, Klinikum rechts der Isar, Technische Universität München (Philips Achieva 3 T TX: *N* = 118; Philips Ingenia 3 T systems: *N* = 21), and the Department of Radiology, University Hospital of Bonn (Philips Achieva 3 T TX: *N* = 16; Philips Ingenia 3 T systems: *N* = 51). An 8-channel sensitivity encoding (SENSE) head coil and identical sequence parameters were employed at all scanners. Regular quality assessments ensured optimal scanning conditions, and MRI physicists at both imaging centers routinely scanned imaging phantoms to monitor within-scanner signal stability over time.

For structural imaging, a high-resolution T1-weighted 3D-Turbo Field Echo sequence was acquired with the following parameters: TR = 7.7ms, TE = 3.9ms, TI=1300ms, flip angle = 15°, parallel acquisition with SENSE factor 2, 180 sagittal slices, field of view=256 × 256 mm, reconstruction matrix = 256 × 256, and an isotropic voxel size of 1 mm³.

### Quality assessment of MRI data

All images were visually inspected for artefacts and underwent quality control. Further, image quality was evaluated using MRIQC Version 24.1.0. using derived contrast-to-noise ratio values to determine outliers exceeding 2 standard deviations from the mean. According to this criterion, 7 of 213 participants (all from the VP/VLBW group) were excluded from final analyses.

### Habenula segmentation

The habenula was manually segmented by three independent raters: two board-certified radiologists (Rater 1, Rater 3) and one trained medical student (Rater 2). Manual segmentation was performed by following the anatomical guidelines provided by Lawson et al.^39^. Each voxel potentially associated with the habenula was evaluated across the frontal, sagittal, and transverse planes and the right and left habenula were coded separately. Segmentation was performed using ITK-SNAP (Version 3.6.0-beta).

Given the small volume of the habenula, assessing inter-rater reliability was particularly essential. For this purpose, an intraclass correlation coefficient (ICC) and Dice Similarity Coefficients (DSC) were calculated. The ICC is a statistical measure used to quantify inter-rater reliability for repeated measurements of an interval-scaled variable between raters^40^. A two-way mixed-effects model with absolute agreement was used to compute the ICC, ensuring the consideration of potential unknown and systematic errors. To interpret the ICC quality, we relied on Cicchetti^41^. DSC were calculated between all rater pairs (R1 vs. R2, R1 vs. R3, R2 vs. R3) for left and right habenula separately, providing a complementary measure of spatial overlap.

### Relative habenula volume

For the analysis of the habenula volumes, the mean relative habenula volume of all three raters was calculated per participant, to obtain a reliable estimate. Means were calculated for left and right habenula volume separately. Furthermore, the relative habenula volume (mean habenula volume divided by intracranial volume (ICV)) was used in all analyses to account for ICV differences and reduce its potential bias, since preterm individuals tend to have smaller ICVs compared to the TB individuals^42^. ICV was determined using the CAT12 toolbox version 12.7^43^.

### Statistical analyses

Statistical analyses were conducted using IBM SPSS Version 29.0. Analyses were conducted for both the combined VP/VLBW group and the VP subgroup separately. The VP/VLBW group was retained to maximize statistical power and ensure comparability with the broader preterm literature, in which prematurity is defined by both GA and BW. Separate VP subgroup analyses were additionally performed, following explicit recommendations from previous studies^26–28^ that GA represents a more homogeneous grouping criterion than BW.

Normality was assessed for all interval-scaled demographic, psychological, and habenula volume data. For group comparisons, independent t-tests were used for normally distributed data, while Mann-Whitney U tests were applied to non-normally distributed data. Assumptions of homogeneity of variances for the t-tests were assessed using Levene’s test. In cases of significant variance inhomogeneity, as indicated by Levene’s test (*p* < 0.05), group comparisons were conducted using Welch’s t-test. Categorical demographic variables were analyzed using chi-square tests. Effect sizes were calculated using Cohen’s d for interval-scaled data, Cramér’s V for nominal and ordinal data, and the Rank-biserial correlation coefficient (r) for non-parametric tests.

Finally, in case of significant habenula volume effects, correlation analyses examined their relationships with (1) INTI and DNTI; and (2) psychological outcomes (BDI, the YASR DSM-oriented scales for anxiety, depression, and avoidant personality). Spearman’s rank correlation coefficient was used for correlation analyses involving non-normally distributed variables. Additionally, to assess whether the observed pattern was specific to the habenula, we compared relative volumes of other subcortical structures (thalamus, caudate, putamen, pallidum, hippocampus) between VP and TB individuals, using independent t-tests. Volumes were derived from automated segmentation with FastSurfer 2.4.2.

Two test families were defined for the primary analyses and corrected using the Bonferroni method: (1) habenula volume group comparisons, comprising four tests (left and right habenula volume compared separately between VP vs. TB and VP/VLBW vs. TB), and (2) neonatal treatment parameter correlations, comprising four tests (correlations of relative right habenula volume with INTI and DNTI, examined separately in the VP/VLBW and VP groups). Psychological outcome group comparisons were prespecified as secondary, exploratory analyses, consistent with the primary focus of this study on habenula morphology. Given that the observed effects are consistent with prior meta-analytic evidence^3,4^, uncorrected p-values are reported alongside effect sizes for these comparisons. Additionally, supplementary analyses comparing relative volumes of other subcortical structures were conducted to assess structure-specificity of the habenula finding and are reported without correction for multiple comparisons. Statistical significance for all analyses was set at *p* < 0.05.

## Results

### Sample characteristics

The final analyses comprised 96 VP/VLBW and 110 TB individuals. Of the 96 VP/VLBW participants, 76 were classified as VP. This study not only focused on the comparison of VP/VLBW versus TB individuals, but specifically focused on the comparison of the VP versus the TB group. Table 1 presents the demographic and clinical characteristics of the samples.

Comparing the VP/VLBW with the TB group revealed no significance in sex distribution (χ²=0.17, *p* = 0.680, V = 0.03), age at assessment (Z=−1.18, *p* = 0.236, r_rb_=−0.08), and handedness (χ²=0.93, *p* = 0.628, V = 0.07). However, VP/VLBW individuals had a significantly lower GA (Z=−12.50, *p* < 0.001, r_rb_=−0.86), BW (t(195.13)=−39.03, *p* < 0.001, d=−5.33), and smaller ICV (t(184)=−3.46, *p* < 0.001, d=−0.48) compared to TB individuals.

Sub-group analysis comparing the VP group only with the TB group revealed no significance in gender distribution (χ²=0.00, *p* = 1.000, V = 0.00), age at assessment (Z=−1.80, *p* = 0.071, r_rb_=−0.13) and handedness (χ²=2.09, *p* = 0.352, V = 0.11). However, VP individuals had a significantly lower GA (Z=−11.74, *p* < 0.001, r_rb_=−0.85), BW (t(184)=−34.18, *p* < 0.001, d=−5.10), and smaller ICV (t(184)=−3.47, *p* < 0.001, d =−0.52) compared to TB individuals.

**Table 1:** Sample characteristics.

	VP/VLBW	VP	TB
N	96	76	110
Sex (m/f) [N]	54/42	45/31	65/45
Handedness (r/l/a) [N]	82/9/4^†^	67/6/2^†^	90/10/8^††^
Age at MRI examination [years]	26.66 ± 0.60	26.60 ± 0.57	26.80 ± 0.74
GA [weeks]	30.5 ± 2.1	29.7 ± 1.4	39.7 ± 1.1
BW [g]	1323 ± 312	1319 ± 344	3397 ± 446
DNTI [days]	53.08 ± 29.22^†^	56.04 ± 30.06^†^	-
INTI [a.u.]	11.37 ± 3.73^†^	12.04 ± 3.61^†^	-
ICV[mm ^3^]	1422 ± 144	1418 ± 137	1495 ± 155

Data are presented as mean ± standard deviation, or frequency, respectively. a=ambidextrous, a.u.=arbitrary unit, BW=birth weight, DNTI=duration of neonatal treatment index, f=female, GA=gestational age, ICV=intracranial volume, INTI=intensity of neonatal treatment index, l=left-handed, m=male, MRI=magnetic resonance imaging, r=right-handed, TB=term-born, VLBW=very low birth weight, VP=very preterm; ^†^ 1 missing dataset, ^† †^ 2 missing datasets.

### Habenula segmentation

Absolute habenula volumes per rater and the mean across raters were comparable to segmented habenula volumes in previous literature^45–49^ and are presented in Supplemental Material Table S1. Inter-rater reliability showed good [0.60–0.74 according to Cicchetti^41^ agreement. In the total sample, the ICC was 0.650 (95%CI [0.467–0.761]), for the left habenula 0.642 (95%CI [0.540–0.723]), and for the right habenula 0.609 (95%CI [0.357–0.747]). ICC values for left and right habenula assessment for each sub-group separately (VP, VP/VLBW, and TB) are presented in Supplementary Material Table S2.

Complementary DSC calculations revealed a mean inter-rater bilateral overlap of 0.735 across all rater pairs (right habenula DSC = 0.738, left habenula DSC = 0.731), with all pairwise DSC values exceeding 0.70, as shown in Supplementary Material Table S3.

### Relative habenula volume

Comparing the VP/VLBW with the TB group revealed no significant differences in left or right relative habenula volume (for statistics see Supplementary Material Table S4).

**Fig. 1:**
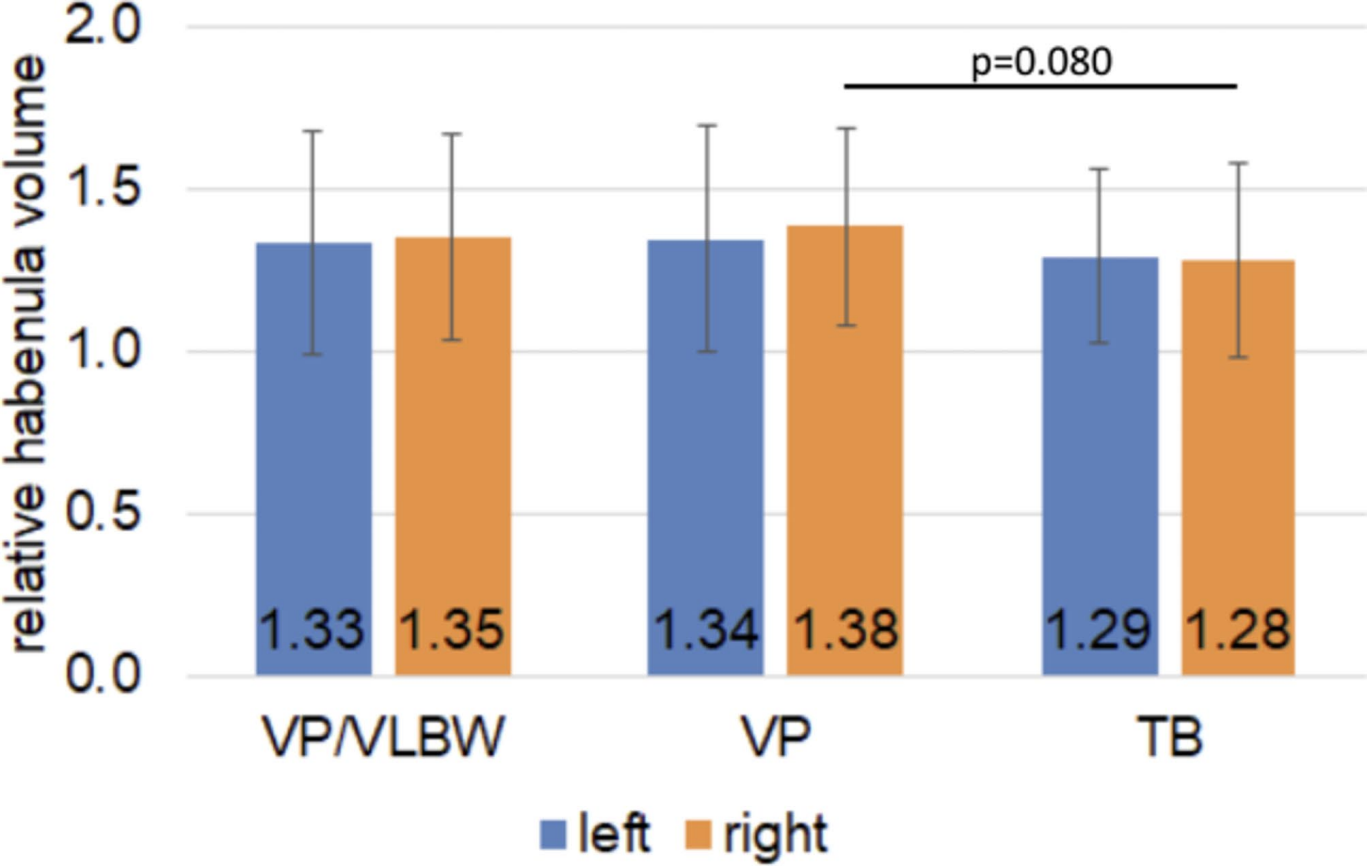
Left and right relative habenula volumes for each cohort (VP/VLBW, VP and TB) TB=term-born, VLBW=very low birth weight, VP=very preterm; *p* = 0.080 (Bonferroni-corrected).

Comparing the VP with the TB group revealed a significant difference for the relative right habenula volume, showing larger relative volumes in the VP compared to TB individuals (t(184) = 2.35, *p* = 0.020, d = 0.351). This difference did not survive Bonferroni correction across the family of habenula volume group comparisons (Bonferroni-corrected *p* = 0.080) and is therefore interpreted as a statistical trend. No significant differences were found for relative left habenula volume (t(184) = 1.13, *p* = 0.261, d = 0.168). Mean relative habenula volumes are presented in Fig. 1.

In an additional sensitivity analysis, an analysis of covariance with scanner site as a covariate observed a similar difference in relative right habenula volume in VP vs. TB individuals (F(1, 183) = 4.17, *p* = 0.042 uncorrected), suggesting that the observed group effect is not solely driven by scanner-related variance, which was not a significant predictor in this model (F(1, 183) = 2.31, *p* = 0.13). Yet, the higher p-value for the group effect did not survive stringent Bonferroni correction.

Correlations between relative habenula volume and neonatal treatment.

**Fig. 2:**
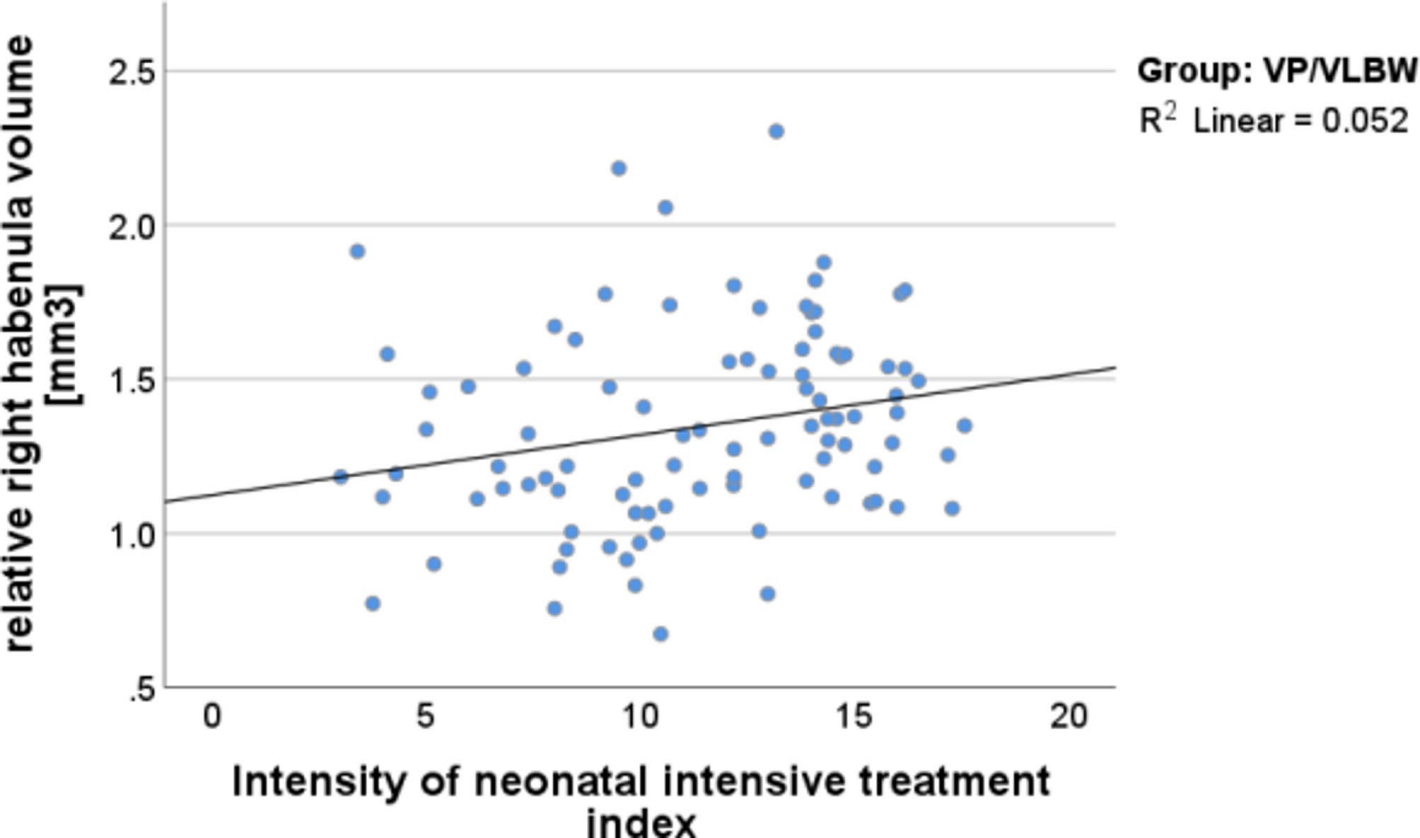
Correlation of relative right habenula volume with Intensity of neonatal treatment index for the VP/VLBW group. VLBW=very low birth weight, VP=very preterm.

A significant correlation was found between relative right habenula volume and INTI when investigating VLBW and VP together (r_s_=0.258, *p* = 0.012, Fig. 2), remaining significant after Bonferroni correction across the family of neonatal treatment parameter correlations (Bonferroni-corrected *p* = 0.048). For the VP only, no significant correlation was found. Furthermore, no significant correlation was found between relative habenula volume and DNTI (see Supplementary Material Table S5 for statistics).

### Affective symptoms in adulthood

Table 2 shows the psychological outcomes of the samples.

Comparing the VP/VLBW with the TB group revealed significant differences for YASR DSM-oriented scale for anxiety (Z=−2.14, *p* = 0.032, r_rb_=−0.15), and YASR DSM-oriented scale for avoidant personality (Z=−2.36, *p* = 0.018, r_rb_=−0.16). BDI (Z=−0.54, *p* = 0.592, r_rb_=−0.04) and YASR DSM-oriented scale for depression (Z=−0.96, *p* = 0.338, r_rb_=−0.07) showed no significant differences between groups.

Sub-group analysis comparing the VP group only with the TB group revealed significant differences for the YASR DSM-oriented scale for avoidant personality (Z=−2.41, *p* = 0.016, r_rb_=−0.17), while the YASR DSM-oriented scale for anxiety revealed a strong trend (Z=−1.95, *p* = 0.051, r_rb_=−0.14). BDI (Z=−0.65, *p* = 0.518, r_rb_=−0.05) and YASR DSM-oriented scale for depression (Z=−1.17, *p* = 0.241, r_rb_=−0.09) showed no significant differences between groups.

**Table 2:** Psychological outcomes.

	VP/VLBW	VP	TB
BDI	3.96 ± 5.08^†^	4.20 ± 5.43^†^	3.20 ± 4.28^††^
YASR DSM-oriented scale for depression	54.05 ± 7.15 ^††^	54.27 ± 7.40 ^††^	52.99 ± 5.32
YASR DSM-oriented scale for anxiety	52.14 ± 4.21 ^††^	52.19 ± 4.50 ^††^	51.15 ± 2.88
YASR DSM-oriented scale for avoidant personality	55.80 ± 8.25 ^† †^	55.72 ± 8.12 ^† †^	52.66 ± 5.42

Data are presented as mean ± standard deviation. BDI=Beck Depression Inventory, TB=term-born, VLBW=very low birth weight, VP=very preterm, YASR=Young Adult Self Report; ^†^1 missing dataset, ^† †^ 2 missing datasets.

Correlations between relative habenula volume and psychological symptoms.

No significant correlations were found between relative habenula volumes and psychological symptoms (see Supplementary Material Table S5 for statistics).

### Relative volumes of subcortical structures

Relative right and left volumes of all examined subcortical structures were significantly reduced in VP compared to TB individuals (all *p* ≤ 0.038), with effect sizes ranging from d = 0.31–1.56. The most pronounced reductions were observed for the thalamus (left: *p*<0.001, d = 1.562; right: *p*<0.001, d = 1.556) and hippocampus (left: *p*<0.001, d = 0.773; right: *p*<0.001, d = 0.913), while putamen and pallidum showed smaller but significant reductions (d = 0.31–0.44). Full results are presented in Supplementary Table S6.

## Discussion

This study is the first to examine habenular volume in VP/VLBW adults, revealing a trend towards a higher relative volume of the right habenula compared to TB controls in the subgroup of the VP-born participants, which did not survive Bonferroni correction (*p* = 0.080). Within the VP/VLBW sample we also found a positive correlation with intensity but not duration of neonatal intensive care. In addition, VP/VLBW and VP-only participants scored significantly higher on standardized measures of anxiety and avoidant personality traits than TB individuals. Notably, we did not observe significant correlations between relative habenular volume and psychological measures.

The habenula, despite its small size, is a key structure for monoaminergic modulation^50,51^ and has attracted growing interest in mood disorders due to its functional relevance in the reward system^9,52,53^. Yet, to date, the habenula has not been studied in the context of preterm birth. Recent research further implicates the habenula in the pathophysiology of neuropsychiatric conditions such as depression and anxiety^20,54^. While the hypothesis of habenula hyperactivity in depression is supported by several functional imaging and animal studies^23,55–58^, reported MRI findings have been inconsistent regarding habenula size changes across different clinical populations^18^. Our measured absolute habenula volumes are congruent to the reported range of published in vivo MRI studies (15–25 mm³ per hemisphere)^45–49^.

Inter-rater reliability was assessed using both ICC and DSC: the overall ICC of 0.650 falls within Cicchetti’s^41^ range of good agreement, and the mean inter-rater DSC of 0.735 can be considered good when compared with values reported for the habenula^46^ and other small subcortical structures^59–61^. Both metrics together support the validity of the habenula segmentation approach, particularly given the exceptionally small size of the structure and the standard 1 mm³ voxel resolution employed.

The trend towards a higher relative right habenula volume emerged only in the VP subgroup, not in the broader VP/VLBW group. This may be explained by increased variance introduced by including VLBW-only individuals, who represent a more heterogeneous population, often including a higher proportion of small-for-gestational-age births and differing somatic and perinatal risk profiles^26–28^, potentially obscuring subtle structural differences such as those observed in relative habenula volume.

While the predominant pattern in preterm individuals involves generally reduced brain volumes, frequently observed in regions such as the thalamus, hippocampus, amygdala, corpus callosum, and basal ganglia^5,42,62^, our findings show a trend towards a relatively enlarged right habenula of participants with preterm GA, after taking their lower ICV into account. Consistent with the pattern of widespread reductions, the present analyses confirmed significantly reduced relative volumes of all examined adjacent subcortical structures (thalamus, caudate, putamen, pallidum, hippocampus) in VP compared to TB individuals. The trend towards relative enlargement of the right habenula therefore cannot be attributed to a global ICV effect but suggests a structure-specific pattern.

Notably, prior analyses in this^63^, and other cohorts^64,65^, have demonstrated that preterm birth can be associated with bidirectional region-specific volumetric effects, including focal grey matter increases alongside widespread reductions, suggesting that region-specific enlargements are not without precedent in preterm populations.

While GA is one possible factor, other parameters such as medical complications (e.g., hypoxia, infections) may also contribute to relative habenula volume variations, as suggested by the positive correlation between relative right habenula volume and INTI across the VP/VLBW subsample.

Our psychological findings are consistent with a well-established body of literature on mental health outcomes in preterm-born populations. Elevated anxiety levels and avoidant personality traits among VP/VLBW individuals align with previous studies, as summarized in a meta-analysis by Fitzallen et al.^3^, which reported significantly higher odds of anxiety disorders, particularly generalized anxiety disorder and specific phobias. The same meta-analysis found no significant increase in depressive disorders, being in line with the results of this study. Likewise, the elevated scores for avoidant personality traits in our study align with findings from a meta-analysis by Pyhälä et al.^4^, which also utilized the YASR scales. However, no significant correlations were found when correlating these psychological outcomes with relative habenula volume, not confirming our a priori assumptions. This concurs with earlier negative findings for the amygdala^8^. In fact, previous studies tended to claim associations between aversive processing and affective disorders and left habenular function: The observed psychological traits may therefore arise from complex, multifactorial influences beyond measurable habenular volume alone^66^.

While our study offers novel insights into habenular structure in preterm-born adults, several limitations must be acknowledged. The sample size may limit statistical power and generalizability, and the present findings should be considered exploratory, pending replication in larger samples. Furthermore, the underlying neurobiological mechanism of the observed trend (e.g., selective preservation, compensatory reorganization, or disproportionate reduction of surrounding structures) cannot be determined from the present data. Additionally, the lateralization of the trend in relative right habenula volume remains difficult to interpret and requires replication in independent samples. Moreover, the MRI data were acquired with a voxel resolution of 1 mm³, which may not fully capture the subtle habenular details given its small size and acquired in a multi-scanner setting which did not have a systematic effect, but increased overall variance.

Manual segmentation carries inherent risks of inter-rater variability. Although the overall ICC of 0.650 falls within Cicchetti’s^41^ range of good agreement and complementary DSC analyses indicated good spatial overlap (mean DSC 0.735), subgroup-specific ICC values varied between 0.573 (left habenula, TB group) and 0.730 (left habenula, VP group), which should be considered when interpreting the present findings. Lastly, as an observational study, our findings are correlational and do not allow for causal inference regarding the effects of preterm birth on brain structure or psychological outcomes. These limitations emphasize the need for careful interpretation and point to areas that warrant further investigation.

## Conclusion

This is the first human study to investigate habenular morphology in adults born very preterm. The finding of a trend towards an enlarged relative right habenula is both novel and unexpected, contrasting with the general trend of volumetric reductions in preterm populations. In the absence of functional or connectivity data, these findings should be interpreted as hypothesis-generating with respect to habenula involvement in affective vulnerability following preterm birth. Future studies with larger sample sizes, high-resolution MRI, and functional imaging approaches are needed to replicate these findings and clarify whether the observed structural alterations reflect changes in habenula function and/or connectivity.

## Supplementary Information

Below is the link to the electronic supplementary material.

Supplementary Material 1

Supplementary Material 2

## Data Availability

Patient data used in this study are not publicly available but stored by the principal investigators of the Bavarian Longitudinal Study.

## References

[1] World Health Organization, Preterm birth. (2023). https://www.who.int/news-room/fact-sheets/detail/preterm-birth, accessed 31 March 2025.

[2] Anderson, J. G. et al. Survival and major morbidity of extremely preterm infants: A population-based study. *Pediatrics*10.1542/peds.2015-4434 (2016). 27302979 10.1542/peds.2015-4434

[3] Fitzallen, G. C. et al. Anxiety and Depressive Disorders in Children Born Preterm: A Meta-Analysis. *J. Dev. Behav. Pediatr. JDBP*. **42**, 154–162. 10.1097/DBP.0000000000000898 (2021). 33480635 10.1097/DBP.0000000000000898

[4] Pyhälä, R. et al. Self-reported mental health problems among adults born preterm: A meta-analysis. *Pediatrics*10.1542/peds.2016-2690 (2017). 28283612 10.1542/peds.2016-2690

[5] Kelly, C. E. et al. Long-lasting effects of very preterm birth on brain structure in adulthood: A systematic review and meta-analysis. *Neurosci. Biobehav. Rev.* **147**, 105082. 10.1016/j.neubiorev.2023.105082 (2023). 36775083 10.1016/j.neubiorev.2023.105082

[6] Groenewold, N. A. et al. Volume of subcortical brain regions in social anxiety disorder: mega-analytic results from 37 samples in the ENIGMA-Anxiety Working Group. *Mol. Psychiatry*. **28**, 1079–1089. 10.1038/s41380-022-01933-9 (2023). 36653677 10.1038/s41380-022-01933-9 PMC10804423

[7] Gray, J. P. et al. Multimodal Abnormalities of Brain Structure and Function in Major Depressive Disorder: A Meta-Analysis of Neuroimaging Studies. *Am. J. Psychiatry*. **177**, 422–434. 10.1176/appi.ajp.2019.19050560 (2020). 32098488 10.1176/appi.ajp.2019.19050560 PMC7294300

[8] Schmitz-Koep, B. et al. Decreased amygdala volume in adults after premature birth. *Sci. Rep.* **11**5403. 10.1038/s41598-021-84906-2 (2021). 33686187 10.1038/s41598-021-84906-2 PMC7970879

[9] Matsumoto, M. & Hikosaka, O. Lateral habenula as a source of negative reward signals in dopamine neurons. *Nature* **447**, 1111–1115. 10.1038/nature05860 (2007). 17522629 10.1038/nature05860

[10] Hong, S. et al. Negative reward signals from the lateral habenula to dopamine neurons are mediated by rostromedial tegmental nucleus in primates. *J. Neurosci. official J. Soc. Neurosci.* **31**, 11457–11471. 10.1523/JNEUROSCI.1384-11.2011 (2011). 10.1523/JNEUROSCI.1384-11.2011 PMC3315151 21832176

[11] Shelton, L. et al. Unmasking the mysteries of the habenula in pain and analgesia. *Prog. Neurobiol.* **96**, 208–219. 10.1016/j.pneurobio.2012.01.004 (2012). 22270045 10.1016/j.pneurobio.2012.01.004 PMC3465722

[12] Ogawa, S. & Parhar, I. S. Functions of habenula in reproduction and socio-reproductive behaviours. *Front. Neuroendocrinol.* **64**, 100964. 10.1016/j.yfrne.2021.100964 (2022). 34793817 10.1016/j.yfrne.2021.100964

[13] Mendoza, J. Circadian neurons in the lateral habenula: Clocking motivated behaviors. *Pharmacol. Biochem. Behav.* **162**, 55–61. 10.1016/j.pbb.2017.06.013 (2017). 28666896 10.1016/j.pbb.2017.06.013

[14] Baño-Otálora, B. (ed ) Contributions of the lateral habenula to circadian timekeeping. *Pharmacol. Biochem. Behav.* **162** 46–54 10.1016/j.pbb.2017.06.007 (2017). 28624585 10.1016/j.pbb.2017.06.007

[15] Hikosaka, O. The habenula: From stress evasion to value-based decision-making. *Nature reviews. Neuroscience* **11**, 503–513. 10.1038/nrn2866 (2010). 20559337 10.1038/nrn2866 PMC3447364

[16] Hennigan, K. et al. Distinct midbrain and habenula pathways are involved in processing aversive events in humans. *J. Neurosci. official J. Soc. Neurosci.* **35**, 198–208. 10.1523/JNEUROSCI.0927-14.2015 (2015). 10.1523/JNEUROSCI.0927-14.2015 PMC4287142 25568114

[17] Lawson, R. P. et al. The habenula encodes negative motivational value associated with primary punishment in humans. *Proc. Natl. Acad. Sci. U.S.A.* **111**, 11858–11863. 10.1073/pnas.1323586111 (2014). 25071182 10.1073/pnas.1323586111 PMC4136587

[18] Fakhoury, M. The habenula in psychiatric disorders: More than three decades of translational investigation. *Neurosci. Biobehav. Rev.* **83**, 721–735. 10.1016/j.neubiorev.2017.02.010 (2017). 28223096 10.1016/j.neubiorev.2017.02.010

[19] Proulx, C. D. et al. Reward processing by the lateral habenula in normal and depressive behaviors. *Nat. Neurosci.* **17**, 1146–1152. 10.1038/nn.3779 (2014). 25157511 10.1038/nn.3779 PMC4305435

[20] Yang, Y. et al. Lateral habenula in the pathophysiology of depression. *Curr. Opin. Neurobiol.* **48**, 90–96. 10.1016/j.conb.2017.10.024 (2018). 29175713 10.1016/j.conb.2017.10.024

[21] Roman, E. et al. Untangling the dorsal diencephalic conduction system: a review of structure and function of the stria medullaris, habenula and fasciculus retroflexus. *Brain Struct. function*. **225**, 1437–1458. 10.1007/s00429-020-02069-8 (2020). 10.1007/s00429-020-02069-8 32367265

[22] Hétu, S. et al. Asymmetry in functional connectivity of the human habenula revealed by high-resolution cardiac-gated resting-state imaging. *Hum. Brain. Mapp.* **37**, 2602–2615. 10.1002/hbm.23199 (2016). 27038008 10.1002/hbm.23194 PMC4905773

[23] Liu, W. H. et al. Association between habenula dysfunction and motivational symptoms in unmedicated major depressive disorder. *Soc. Cognit. Affect. Neurosci.* **12**, 1520–1533. 10.1093/scan/nsx018 (2017). 28575424 10.1093/scan/nsx074 PMC5629818

[24] Yoshino, A. et al. Importance of the habenula for avoidance learning including contextual cues in the human brain: A preliminary fMRI study. *Front. Hum. Neurosci.* **14**, 165. 10.3389/fnhum.2020.00165 (2020). 32477084 10.3389/fnhum.2020.00165 PMC7235292

[25] Abuduaini, Y. et al. Significant heterogeneity in structural asymmetry of the habenula in the human brain: A systematic review and meta-analysis. *Hum. Brain. Mapp.* **44**, 4165–4182. 10.1002/hbm.26337 (2023). 37195040 10.1002/hbm.26337 PMC10258539

[26] Arnold, C. C. et al. Very low birth weight: A problematic cohort for epidemiologic studies of very small or immature neonates. *Am. J. Epidemiol.* **134**, 604–613. 10.1093/oxfordjournals.aje.a116133 (1991). 1951265 10.1093/oxfordjournals.aje.a116133

[27] Koller-Smith, L. I. et al. Comparing very low birth weight versus very low gestation cohort methods for outcome analysis of high risk preterm infants. *BMC Pediatr.* **17**, 166. 10.1186/s12887-017-0921-x (2017). 28709451 10.1186/s12887-017-0921-x PMC5512978

[28] Hollanders, J. J. et al. Long-term neurodevelopmental and functional outcomes of infants born very preterm and/or with a very low birth weight. *Neonatology* **115**, 310–319. 10.1159/000495133 (2019). 30836372 10.1159/000495133 PMC6604264

[29] Riegel *Die Entwicklung gefährdet geborener Kinder bis zum fünften Lebensjahr: Die Arvo-Ylppö-Neugeborenen-Nachfolgestudie in Südbayern und Südfinnland; 63 Tabellen* (Enke, 1995).

[30] Wolke, D. et al. The cognitive outcome of very preterm infants may be poorer than often reported: an empirical investigation of how methodological issues make a big difference. *Eur. J. Pediatrics*. **153**, 906–915. 10.1007/BF01954744 (1994). 10.1007/BF01954744 7532133

[31] Breeman, L. D. et al. Preterm cognitive function into adulthood. *Pediatrics* **136**, 415–423. 10.1542/peds.2015-0608 (2015). 26260714 10.1542/peds.2015-0608

[32] Dubowitz, L. M. et al. Clinical assessment of gestational age in the newborn infant. *J. Pediatr.* **77**, 1–10. 10.1016/s0022-3476(70)80038-5 (1970). 5430794 10.1016/s0022-3476(70)80038-5

[33] Gutbrod, T. et al. Effects of gestation and birth weight on the growth and development of very low birthweight small for gestational age infants: A matched group comparison. *Arch. Dis. Child. Fetal Neonatal Ed.* **82**, F208-14. 10.1136/fn.82.3.f208 (2000). 10794788 10.1136/fn.82.3.F208 PMC1721075

[34] Dragovic, M. Categorization and validation of handedness using latent class analysis. *Acta neuropsychiatrica*. **16**, 212–218. 10.1111/j.0924-2708.2004.00087.x (2004). 26984309 10.1111/j.0924-2708.2004.00087.x

[35] Oldfield, R. C. The assessment and analysis of handedness: the Edinburgh inventory. *Neuropsychologia* **9**, 97–113. 10.1016/0028-3932(71)90067-4 (1971). 5146491 10.1016/0028-3932(71)90067-4

[36] Achenbach, T. M. *Manual for the Young Adult Self Report and Young Adult Behavior Checklist* (University of Vermont, Department of Psychiatry, 1997).

[37] Ni, Y. et al. Bullying victimisation in childhood and mental health in early adulthood: comparison of prospective and retrospective reports. *Curr. Psychol.* **43**, 19666–19675. 10.1007/s12144-024-05788-x (2024).

[38] Beck, A. T. An inventory for measuring depression. *Arch. Gen. Psychiatry* **4**, 561–571. 10.1001/archpsyc.1961.01710120031004 (1961). 13688369 10.1001/archpsyc.1961.01710120031004

[39] Lawson, R. P. et al. Defining the habenula in human neuroimaging studies. *NeuroImage* **64**, 722–727. 10.1016/j.neuroimage.2012.08.076 (2013). 22986224 10.1016/j.neuroimage.2012.08.076 PMC3650642

[40] Shrout, P. E. & Fleiss, J. L. Intraclass correlations: uses in assessing rater reliability. *Psychol. Bull.* **86**, 420–428. (1979). 18839484 10.1037//0033-2909.86.2.420

[41] Cicchetti, D. V. Guidelines, criteria, and rules of thumb for evaluating normed and standardized assessment instruments in psychology. *Psychol. Assess.* **6**, 284–290. 10.1037/1040-3590.6.4.284 (1994).

[42] Kuula, J. et al. Brain volumes and abnormalities in adults born preterm at very low birth weight. *J. Pediatr.* **246**, 48-55.e7. 10.1016/j.jpeds.2022.03.009 (2022). 35301016 10.1016/j.jpeds.2022.03.009

[43] Gaser, C. et al. CAT: a computational anatomy toolbox for the analysis of structural MRI data. *GigaScience* 13. 10.1093/gigascience/giae049 (2024). 10.1093/gigascience/giae049 PMC11299546 39102518

[44] Henschel, L. et al. FastSurfer – A fast and accurate deep learning based neuroimaging pipeline. *Neuroimage* **219**, 117012. 10.1016/j.neuroimage.2020.117012 (2020). 32526386 10.1016/j.neuroimage.2020.117012 PMC7898243

[45] Cho, S.-E. et al. Left-right asymmetric and smaller right habenula volume in major depressive disorder on high-resolution 7-T magnetic resonance imaging. *PLoS One* **16**, e0255459. 10.1371/journal.pone.0255459 (2021). 34343199 10.1371/journal.pone.0255459 PMC8330903

[46] Kim, J. W. et al. Reproducibility of myelin content-based human habenula segmentation at 3 Tesla. *Hum. Brain. Mapp.* **39**, 3058–3071. 10.1002/hbm.24060 (2018). 29582505 10.1002/hbm.24060 PMC6033622

[47] Lawson, R. P. et al. Disrupted habenula function in major depression. *Mol. Psychiatry*. **22**, 202–208. 10.1038/mp.2016.81 (2017). 27240528 10.1038/mp.2016.81 PMC5285459

[48] Savitz, J. B. et al. Habenula volume in post-traumatic stress disorder measured with high-resolution MRI. *Biology of mood & anxiety disorders* **1**, 7. 10.1186/2045-5380-1-7 (2011). 22738208 10.1186/2045-5380-1-7 PMC3384261

[49] Schmidt, F. M. et al. Habenula volume increases with disease severity in unmedicated major depressive disorder as revealed by 7T MRI. *Eur. Arch. Psychiatry Clin. NeuroSci.* **267**, 107–115. 10.1007/s00406-016-0675-8 (2017). 26873703 10.1007/s00406-016-0675-8

[50] Hu, H. et al. Circuits and functions of the lateral habenula in health and in disease. *Nat. Rev. Neurosci.* **21**, 277–295. 10.1038/s41583-020-0292-4 (2020). 32269316 10.1038/s41583-020-0292-4

[51] Viswanath, H. et al. The medial habenula: still neglected. *Front. Hum. Neurosci.* **7**, 931. 10.3389/fnhum.2013.00931 (2013). 24478666 10.3389/fnhum.2013.00931 PMC3894476

[52] Bromberg-Martin, E. S. & Hikosaka, O. Lateral habenula neurons signal errors in the prediction of reward information. *Nat. Neurosci.* **14**, 1209–1216. 10.1038/nn.2902 (2011). 21857659 10.1038/nn.2902 PMC3164948

[53] Tian, J. & Uchida, N. Habenula Lesions Reveal that Multiple Mechanisms Underlie Dopamine Prediction Errors. *Neuron* **87**, 1304–1316. 10.1016/j.neuron.2015.08.028 (2015). 26365765 10.1016/j.neuron.2015.08.028 PMC4583356

[54] Zhang, G.-M. et al. Multi-level variations of lateral habenula in depression: A comprehensive review of current evidence. *Front. Psychiatry* **13**, 1043846. 10.3389/fpsyt.2022.1043846 (2022). 36386995 10.3389/fpsyt.2022.1043846 PMC9649931

[55] Li, K. et al. βCaMKII in lateral habenula mediates core symptoms of depression. *Science* **341**, 1016–1020. 10.1126/science.1240729 (2013). 23990563 10.1126/science.1240729 PMC3932364

[56] Sartorius, A. et al. Remission of major depression under deep brain stimulation of the lateral habenula in a therapy-refractory patient. *Biol. Psychiatry*. **67**, e9–e11. 10.1016/j.biopsych.2009.08.027 (2010). 19846068 10.1016/j.biopsych.2009.08.027

[57] Shabel, S. J. et al. Mood regulation. GABA/glutamate co-release controls habenula output and is modified by antidepressant treatment. *Sci. (New York N Y)*. **345**, 1494–1498. 10.1126/science.1250469 (2014). 10.1126/science.1250469 PMC4305433 25237099

[58] Winter, C. et al. Pharmacological inhibition of the lateral habenula improves depressive-like behavior in an animal model of treatment resistant depression. *Behav. Brain Res.* **216**, 463–465. 10.1016/j.bbr.2010.07.034 (2011). 20678526 10.1016/j.bbr.2010.07.034

[59] Quattrini, L. et al. A test–retest reliability study of automated segmentation of the human habenula at 3T MRI . *NeuroImage*10.1016/j.neuroimage.2020.116932 (2020). 32416226 10.1016/j.neuroimage.2020.116932

[60] Deeley, Q. et al. Hippocampal and subcortical volumetry using atlas-based segmentation of MRI: Validation and comparison with manual delineation. *Phys. Med. Biol.* **56**, 4509–4525. 10.1088/0031-9155/56/14/021 (2011).

[61] Hashempour, N. et al. Effect of different MRI acquisition and processing strategies on subcortical brain structure segmentation. *Front. NeuroSci.* **13**, 1025. 10.3389/fnins.2019.01025 (2019). 31616245 10.3389/fnins.2019.01025 PMC6768976

[62] Inder, T. E. et al. Preterm brain injury. *N. Engl. J. Med.* **389**, 2141–2158. 10.1056/NEJMra2303347 (2023).

[63] Meng, C. et al. Extensive and interrelated subcortical white and gray matter alterations in preterm-born adults. *Brain Struct. Funct.* **221**, 2109–2121. 10.1007/s00429-015-1032-9 (2016). 25820473 10.1007/s00429-015-1032-9

[64] Nosarti, C. et al. Grey and white matter distribution in very preterm adolescents mediates neurodevelopmental outcome. *Brain* **131**, 205–217. 10.1093/brain/awm282 (2008). 18056158 10.1093/brain/awm282

[65] Nosarti, C. et al. Preterm birth and structural brain alterations in early adulthood. *Neuroimage Clin.* **6**, 180–191. 10.1016/j.nicl.2014.08.005 (2014). 25379430 10.1016/j.nicl.2014.08.005 PMC4215396

[66] Martin, E. I. et al. The neurobiology of anxiety disorders: Brain imaging, genetics, and psychoneuroendocrinology. *Psychiatr. Clin. North Am.* **32**, 549–575. 10.1016/j.psc.2009.05.004 (2009). 19716990 10.1016/j.psc.2009.05.004 PMC3684250

