# Supplementary material for: Habenula volume alterations in adults born very preterm
Source: Sci Rep. 2026 Jul 14;16:22071. doi: 10.1038/s41598-026-61917-5 (PMC13369503; doi:10.1038/s41598-026-61917-5)
Supplement: Supplementary file 1 — Supplementary Material 1 [file 41598_2026_61917_MOESM1_ESM.docx]

**Supplemental Material to**

**Habenula Volume Alterations in Adults Born Very Preterm**

Stüwe and Maurer et al.

**Table S1: Absolute Habenula Volumes per Rater**

| Hemisphere | Rater | VP/VLBW | VP | TB |
| --- | --- | --- | --- | --- |
| left | 1 | 19.90 ± 6.87 | 20.25 ± 6.81 | 20.84 ± 5.75 |
|  | 2 | 18.95 ± 5.24 | 19.12 ± 5.24 | 18.81 ± 5.14 |
|  | 3 | 17.73 ± 4.87 | 17.36 ± 5.06 | 17.83 ± 4.48 |
|  |  |  |  |  |
| right | 1 | 21.46 ± 6.35 | 22.34 ± 6.05 | 21.46 ± 6.47 |
|  | 2 | 19.75 ± 4.58 | 20.08 ± 4.52 | 19.71 ± 5.56 |
|  | 3 | 15.73 ± 4.33 | 15.89 ± 4.17 | 15.65 ± 4.33 |

Data are presented as mean ± standard deviation. TB=term-born, VLBW=very low birth weight, VP=very preterm.

**Intraclass Correlation Coefficients**

ICCs were additionally calculated within each sub-group. The highest agreement was observed in the VP group for the left habenula (ICC=0.730, 95% CI [0.598–0.822]), and the lowest in the left habenula of the TB group (ICC=0.573, 95% CI [0.410–0.696]).

**Table S2: Intraclass Correlation Coefficient of Habenula Volume Assessment**

|  | VP/VLBW | VP | TB |
| --- | --- | --- | --- |
| Left | 0.702 (0.580 - 0.792) | 0.730 (0.598 - 0.822) | 0.573 (0.410 - 0.696) |
| Right | 0.620 (0.333 - 0.773) | 0.597 (0.243 - 0.774) | 0.601 (0.344 - 0.749) |

Values are presented as intraclass correlation coefficients (ICC) with 95% confidence intervals. TB=term-born, VLBW=very low birth weight, VP=very preterm.

**Table S3: Comparing relative Habenula Volume in VLBW/VP and TB**

| **Hemisphere** | **Test** | **df** | **Test statistic** | ***p*-value** | **Effect size** |
| --- | --- | --- | --- | --- | --- |
| **left** | Mann–Whitney U | – | *Z*=-0.576 | 0.564 | r_rb_=- 0.04 |
| **right** | t-test | 204 | *t*=1.648 | 0.101 | d=0.230 |

A Mann–Whitney U test was conducted for the left habenula due to non-normally distributed data; an independent t-test was used for the right habenula volume. d=Cohen's d, df=degrees of freedom, r_rb_=rank-biserial correlation coefficient, TB=term-born, VLBW=very low birth weight, VP=very preterm.

**Table S4: Correlations between relative right habenula volume and psychological symptoms and neonatal treatment**

|  | **VLBW/VP and TB** | | **VP and TB** | | **VLBW/VP** | | **VP** | | **TB** | |
| --- | --- | --- | --- | --- | --- | --- | --- | --- | --- | --- |
|  | rₛ | **p-value** | rₛ | **p-value** | rₛ | **p-value** | rₛ | **p-value** | rₛ | **p-value** |
| **BDI** | -0.001 | 0.991 | -0.028 | 0.702 | -0.001 | 0.989 | -0.070 | 0.550 | -0.037 | 0.702 |
| **YASR DSM-oriented scale for depression** | -0.104 | 0.139 | -0.141 | 0.056 | -0.054 | 0.603 | -0.148 | 0.209 | -0.172 | 0.072 |
| **YASR DSM-oriented scale for anxiety** | -0.065 | 0.359 | -0.113 | 0.125 | -0.034 | 0.745 | -0.144 | 0.221 | -0.145 | 0.131 |
| **YASR DSM-oriented scale for avoidant personality** | 0.040 | 0.567 | -0.002 | 0.979 | 0.051 | 0.628 | -0.052 | 0.658 | -0.019 | 0.844 |
| **DNTI [days]** | - | - | - | - | 0.123 | 0.236 | 0.096 | 0.413 | - | - |
| **INTI** | - | - | - | - | 0.258 | 0.012 | 0.163 | 0.163 | - | - |

BDI=Beck Depression Inventory, DNTI=Duration of neonatal treatment index, INTI=Intensity of neonatal treatment index, *rₛ*=Spearman’s rank correlation coefficient, TB=term-born, VLBW=very low birth weight, VP=very preterm, YASR=Young Adult Self-Report.
